# Digital Health Research Symposium: Opening Panel Commentary

**DOI:** 10.1016/j.mcpdig.2024.06.002

**Published:** 2024-09-17

**Authors:** Elizabeth Krupinski, Renee Pekmezaris, Amirala S. Pasha

**Affiliations:** Department of Radiology and Imaging Sciences, Emory University, Atlanta, GA; Northwell Health, Manhasset NY; Zucker School of Medicine, Uniondale, NY; Feinstein Institutes for Medical Research, Manhasset, NY; Division of General Internal Medicine, Mayo Clinic, Rochester, MN

### Scalability—Elizabeth Krupinski, PhD

It is important to recognize that there is a difference between scalability and growth. Scalability is the ability of a system (eg, health care organization) to handle an increasing amount of work and/or users without (negatively) impacting performance or quality of service. With scalability, the focus is on maintaining performance as the load increases by optimizing existing resources. Ideally, performance and quality could increase as a program scales. The importance of scalability for digital health is that it ensures reliability and stability during periods of high demand and enhances user experience by preventing downtime or service interruptions. For example, having a telemedicine platform that can seamlessly accommodate a surge in users during a public health crisis without crashing or slowing down is a scalable solution.

Growth refers to the expansion of a system’s capacity and/or size over time. It is often driven by increased demand or usage and focuses on expanding capacity to accommodate more users or features. Growth often involves investments in infrastructure, technology, or personnel rather than relying on existing resources. The importance of growth for digital health is that it allows providers to reach new markets and demographics and enables innovation and improvement of services to meet evolving patient needs. An example would be a telemedicine provider adding new features, services, personnel, or geographical coverage to meet the needs of a growing user base.

Scalability and growth are both essential for the success of digital health programs. Understanding the differences between the 2 helps in strategic planning and resource allocation to ensure long-term viability and success. So, why is scalability important for digital health? The dramatic increase in telehealth during coronavirus clearly not only found its utility and benefits but also highlighted the many challenges with scalability at both large well-resourced institutions and small practices with far fewer resources. In both scenarios, some programs were more successful than others at scaling their services, with those already having some investment and experience with digital health tending to scale more effectively than those without much of an existing footprint.

Perhaps, the greatest challenge we face with scalability (and growth) obviously has a technology component, but that is secondary to the main challenges of integration and culture. There are some studies addressing this but most of the current knowledge and understanding is at the broader level of health care innovations in general. Implementation sciences and its intersection with industrial and organizational psychology is what we really need far more of to truly realize scalability of innovative solutions. The 2 talks in this meeting on this topic nicely illustrate the types of innovative solutions being developed that with the right next steps could truly scale well beyond the laboratory and local implementation levels.

### Patient Engagement—Renee Pekmezaris, PhD

The session began with a definition of patient engagement: a strategy introduced by pharmaceutical and medical device companies to improve patient adherence to treatment protocols during clinical trials and care processes.^1^ The fact that perspectives vary was discussed (clinicians tend to identify patient engagement as “adherence” with medication recommendations and or protocols; patients tend to think of engagement as having a say in their own health care).

The history of patient engagement was discussed in improving patient outcomes (with health care being late to the engagement game compared with other industries). The importance of patient engagement was addressed next. The audience was provided with an example of patient engagement approaches used in a recent Patient-Centered Outcomes Research Institute (PCORI)-funded digital health study that found substantially improved outcomes (Hemoglobin A1c, blood pressure, and self-efficacy) in underserved Latin-X patients with type 2 diabetes for randomized to a culturally congruent telehealth arm at Northwell Health in New York.

### Team Dynamics—Amirala Pasha, DO, JD

Team dynamics refers to the interpersonal relationships, interactions, and processes that motivate and drive teams toward a common goal. It can encompass communication, collaboration, decision-making, task management, and conflict resolution, contributing to the effectiveness, sustainability, and impactfulness of any innovation.^1^ How clinicians interact with one another while optimizing the use of digital health becomes crucial because the health care system continues its transition toward team-based and value-based care models. When team dynamics is incorporated as an essential design element in any health care solution, it has the potential to eliminate inefficiencies and facilitate communication, team function, and team members’ well-being, leading to optimized care teams and creation of high-performing teams. Similarly, if not appropriately considered as a design element early on, it can yield opposite results. Therefore, any digital health solution that addresses or impacts teams must consider team dynamics as a crucial design element.

Previously, the focus of team-based care was mostly on coordination of care in multidisciplinary teams, where multiple team members from different fields provided care to the patient while maintaining their unique professional boundaries. However, as team-based care progresses in the 21st century, there has been a rise of interdisciplinary teams as part of multidisciplinary teams, where clinicians from what is traditionally considered the same discipline, care for patients in a very specialized manner. This could be clinicians within the same discipline but with different levels of licensure or clinicians with different focuses but equivalent levels of education and licensure.^2^ As interdisciplinary teams become part of the standard of care, the interdependence of team members is increased as each clinician on an interdisciplinary team is only responsible for a more limited aspect of care. This shift within team-based care will necessitate more interpersonal interactions, many of which are facilitated by digital health solutions, further highlighting the importance of considering team dynamics within digital health solutions for the future.

Finally, it is crucial to remember that the technology aspect of digital health solutions can only provide functional changes to improve team dynamics. Another essential aspect of team dynamics is cultural.^3^ Any designer of digital health solutions, in addition to addressing team dynamics from a technical standpoint, must also be prepared to address cultural elements of team dynamics, which may be unique to each health system. Otherwise, at best, the digital health solution fails to capture its full potential, and at worst, the solution may fail or deteriorate patient care even if the design is sound from a technical standpoint.

## Potential Competing Interests

The authors report no competing interests.
